# *Bibi ergo sum*: the effects of a placebo and contextual alcohol cues on motivation to drink alcohol

**DOI:** 10.1007/s00213-016-4518-0

**Published:** 2017-01-07

**Authors:** Paul Christiansen, Gareth Townsend, Graeme Knibb, Matt Field

**Affiliations:** 1 Department of Psychological Sciences, University of Liverpool, Liverpool, L69 7ZA UK; 2 UK Centre for Tobacco and Alcohol Studies (UKCTAS), Nottingham, UK

**Keywords:** Alcohol, Anticipated effects, Context, Craving, Priming, Placebo

## Abstract

**Rationale:**

Acute ‘priming’ doses of alcohol reliably increase alcohol-seeking behaviour in social drinkers. However, the effects of the anticipated (rather than pharmacological) effects of alcohol, and their interaction with contextual alcohol cues, are not well understood.

**Objectives:**

This study aims to determine the extent to which an alcohol-placebo drink increases craving, subjective intoxication and beer consumption, while conjointly investigating the impact of contextual alcohol cues.

**Methods:**

On a within-subject basis, 64 undergraduate social drinkers consumed both a placebo (which they believed to contain alcohol) and a control drink (which they knew did not contain alcohol) in different sessions. Participants completed the study procedures in a bar laboratory designed to look like a ‘pub’ or a standard psychology lab containing no alcohol-related cues. Craving (Desires for Alcohol Questionnaire) and subjective intoxication were measured pre- and post-drink, and a bogus taste test to measure ad-lib alcohol consumption was completed at the end of each session.

**Results:**

Compared to the control drink, placebo significantly increased craving, ad-lib consumption and subjective intoxication, regardless of environmental context.

**Conclusions:**

Increased craving and ad-lib alcohol consumption after consuming a priming dose of alcohol is at least partly attributable to the anticipated rather than the pharmacological effects of the priming dose.

**Electronic supplementary material:**

The online version of this article (doi:10.1007/s00213-016-4518-0) contains supplementary material, which is available to authorized users.

## Introduction

The seminal work of Marlatt et al. (1973) demonstrated that the anticipated effects of alcohol can be an important determinant of loss of control over drinking. Specifically, this study revealed that in both alcohol-dependent and social drinkers, the anticipated, but not pharmacological, effects of alcohol led to increased voluntary ad-lib alcohol consumption. Despite this, recent research efforts investigating increased voluntary drinking following an initial dose of alcohol (alcohol priming effect, de Wit 1996) have focussed on the pharmacological rather than anticipated effects of alcohol.

The alcohol priming effect is argued to be central to binge drinking, and understanding the causes of loss of control following an initial drink has implications for controlling excessive alcohol use as well as consequences of heavy episodic drinking such as sexual risk taking and aggression (see Field et al. 2010). Several theoretical models describing alcohol priming have explained this loss of control of behaviour following an initial prime as the result of alcohol-induced increases in incentive motivation and/or impairments in behavioural control, but are silent on what may be significant anticipated effects (e.g. Field et al. 2010; Koob and Le Moal 2001; Wise and Bozarth 1987). However, other theoretical models (e.g. Moss and Albery 2009) place greater emphasis on the role of alcohol expectancy and alcohol myopia in the priming effect. Specifically, Moss and Albery (2009) highlight the importance of the activation of mental representations associated with alcohol consumption by alcohol-related environmental cues, arguing that this activation is sufficient to change behaviour. Indeed, this model describes a *pre-consumption phase* in which the activation of expectancies can increase (or indeed decrease) the accessibility of alcohol-related behavioural responses, before any pharmacological effects of alcohol are experienced. This suggests that a drink perceived to contain alcohol could activate alcohol-related cognitions thereby priming increased alcohol seeking in the absence of any alcohol-induced cognitive impairment.

The majority of recent priming studies have involved the administration of an alcohol drink which participants are informed that it contains alcohol (i.e. expectancy and pharmacological condition) and a placebo drink that is manipulated to smell and taste of alcohol in which participants are told that it contains alcohol (i.e. expectancy only). Differences between conditions can therefore be attributed to pharmacological effects (e.g. Chutuape et al. 1994; de Wit and Chutuape 1993; Fernie et al. 2012; Fillmore and Rush 2001; Rose and Duka 2006; Rose and Grunsell 2008; Rose et al. 2014; Weafer and Fillmore 2008; Weafer and Fillmore 2013). The lack of an additional experimental condition in which participants receive a drink that does not taste of alcohol and are told contains no alcohol (no-expectancy, no-alcohol condition; hereafter referred to as a control condition) means that these studies cannot isolate the purely anticipated effects of alcohol (which requires both placebo and control conditions). Taken further, we argue that these studies (Chutuape et al. 1994; de Wit and Chutuape 1993; Fernie et al. 2012; Fillmore and Rush 2001; Rose and Duka 2006; Rose and Grunsell 2008; Rose et al. 2014; Weafer and Fillmore 2008; Weafer and Fillmore 2013) are not analogous to ‘real world’ drinking, because when an individual consumes alcohol outside of the laboratory, any effect on behaviour will be the result of the cumulative anticipated and pharmacological effects of alcohol.

Only an alcohol-control comparison can reveal the full impact of an alcoholic drink on craving and alcohol consumption. To give one example of a domain in which this distinction may be important, Schoenmakers et al. (2008) found no pharmacological effect of alcohol on automatic alcohol-approach responses based on an alcohol-placebo comparison. However, a subsequent study that included both alcohol, placebo and control conditions, revealed that automatic alcohol-approach responses were sensitive to the anticipated but not the pharmacological effects of alcohol (Christiansen et al. 2013). Similarly, we recently demonstrated that placebo-alcohol can produce significant impairments in inhibitory control in comparison with a control drink (Christiansen et al. 2016), although the dominant view in the literature is that only moderate to high doses of alcohol (>0.6 g/kg) can impair executive cognitive functioning (for a review see Field et al. 2010).

A limited number of studies have investigated the anticipated effects of alcohol, alone and in combination with its pharmacological effects, although these have used different methodologies and have found inconsistent results. An early meta-analysis (Hull and Bond 1986) of the anticipated and pharmacological effects of alcohol concluded that the anticipated effects of alcohol have important behavioural consequences, arguing the belief that one is intoxicated may provide an *excuse* not to inhibit behaviour. However, using balanced placebo designs, (randomly allocating participants to be told they will receive an alcoholic or non-alcoholic drink, despite actual content) both Craig et al. (2009) and Attwood et al. (2009) found pharmacological, but not anticipated, effects of alcohol on emotional perception. With regard to the alcohol priming effect, we (Christiansen et al. 2013) utilised a partially balanced placebo design in which participants received alcoholic, placebo and control drinks. Compared to a control drink, this study revealed a significant effect of placebo on craving but not ad-lib consumption. However, ad-lib consumption was assessed after an hour-long battery of cognitive tasks, by which time when the placebo effect had dissipated. Rose et al. (2013) attempted to disentangle the anticipated from pharmacological effects of alcohol on alcohol craving and found that craving was increased (compared to a control drink) by both alcohol and placebo; however, the increase following alcohol was immediate and then dissipated, whereas it followed the opposite pattern after the placebo. One other study (Leeman et al. 2009) demonstrated that levels of craving following a placebo (but not alcohol) predicted ad-lib consumption. However, due to the design of that study, it is unclear if the placebo resulted in increased alcohol consumption, or whether the correlation between craving and ad-lib consumption would have been observed even if a placebo had not been administered.

A further issue with many previous studies that investigated acute alcohol effects is that those studies were conducted in neutral laboratories (e.g. Christiansen et al. 2013; Fernie et al. 2012; Rose and Duka 2006; Rose and Grunsell 2008), which creates a context that is very different from the settings in which alcohol is typically consumed. This is advantageous when attempting to isolate the pharmacological effects of alcohol, but the anticipated effects of alcohol may be moderated by the context in which a drink is consumed rather than just the perception that alcohol is consumed. Indeed, Moss and Albery (2009) argue that environmental cues can prime alcohol-related behaviours automatically and therefore have a central role in the alcohol priming effect in the absence of any alcohol consumption.

Substance-related contextual cues have been shown to change alcohol-related cognitions (Monk and Heim 2013) and increase craving (for tobacco, Conklin 2006) possibly by activating brain networks that underlie motivation and acquisitive behaviour (Heinz et al. 2009; Moss and Albery 2009). Moreover, expectancy of intoxication (Wall et al. 2001) and ad-lib alcohol consumption (Lau-Barraco and Dunn 2009; Moss et al. 2015) has been found to be increased in bar-like environments. A limited number of studies have investigated the synergistic effects of bar-like environments and alcohol primes on drinking. For example, Wigmore and Hinson (1991) found that a bar environment increased consumption of both placebo and alcoholic drinks in comparison with a standard lab, although some studies have failed to show such effects (Fromme and Dunn 1992). Though a meta-analysis of priming studies (McKay and Schare 1999) revealed no moderating effects of bar-like environments on subjective and behavioural outcome measures following alcohol primes compared to standard labs, this analysis did not differentiate between diverse outcome measures (including sexual arousal, aggression and anxiety). The specific impact of naturalistic environments on alcohol-seeking behaviours is therefore unclear. Based on the findings of Wigmore and Hinson and those regarding the impact of contextual cues (e.g. Lau-Barraco and Dunn 2009; Moss et al. 2015), we predicted that consumption of a placebo in a cue-rich environment would increase craving and voluntary drinking compared to placebo consumed in a standard laboratory.

The failure to consider the anticipated effects of alcohol and the effects of contextual cues is to the detriment of our understanding of the psychological processes involved in alcohol priming effects. In the present study, we investigated the anticipated effects of alcohol on the priming effect by contrasting subjective craving, intoxication and voluntary drinking behaviour following a placebo and a control beverage. To investigate the influence of environmental context, half of the participants completed the study in a standard laboratory and the other half completed the same procedure in a bar laboratory. We hypothesised that (1) the placebo would result in increased craving, beer consumed in a taste test, and subjective intoxication from pre-drink to post-drink, with no significant change in the control condition, and (2) the effect of the placebo on these measures would be moderated by the environmental context, with larger increases in the bar laboratory compared to a standard laboratory.

## Method

### Participants

An opportunity sample of 64 participants (53 female) aged between 18 and 37 years (mean 19.64 ± 3.98) were recruited via word of mouth and intranet advertising from the University of Liverpool. Participants were invited to take part if they self-reported regular consumption of alcohol (at least one alcoholic drink per week). Furthermore, it was made clear in advertisements and the participant information sheet that all participants should regularly drink beer, because tasting beers was a part of the procedure and a dislike of beer would have resulted in little or no beer consumed. Participants received course credit for their participation.

## Design

A mixed experimental design was employed. The between-subject variable was laboratory (bar laboratory, standard laboratory), with participants allocated into between-subject conditions in four counterbalanced blocks. The within-subject variables were drink type and time: All participants completed a placebo session and a control session (completed 1 week apart; with order of conditions counterbalanced), whereas measures of craving and intoxication were taken twice (pre-drink and post-drink). The taste test was completed once at the end of each session.

## Materials

### Drink preparation (based on Christiansen et al. 2013)

The placebo drink consisted of 500 ml chilled lemonade with vodka applied to the rim of the glass; an atomiser was used to spray vodka mist on the surface of the drink. The control drink consisted of 500 ml chilled water only. Participants were informed that the drink was alcoholic in the placebo condition, and that it was non-alcoholic in the control condition.

### Questionnaires

#### Timeline followback (Sobell and Sobell 1990)

The Timeline followback (TLFB) self-report questionnaire was used to assess alcohol consumption. Participants had to estimate the number of alcohol units consumed over the preceding 14 days (one UK unit = 8 g of alcohol).

#### The Alcohol Use Disorders Identification Test (Saunders et al. 1993)

The Alcohol Use Disorders Identification Test (AUDIT) was used to assess hazardous drinking. The AUDIT consists of ten fixed-response questions regarding alcohol consumption and consequences of drinking. Scores on the AUDIT range between 0 and 40 with scores of 8 or above indicating hazardous or harmful alcohol use.

#### Desires for Alcohol Questionnaire—brief version (Love et al. 1998)

The Desires for Alcohol Questionnaire (DAQ) is a 14-item multidimensional alcohol craving scale that yields scores on three different factors of craving: positive and negative reinforcements, strong desires and intentions and mild desires and intentions. Scores on each factor range from 1 to 7, with higher scores indicative of higher craving. Subsequent analyses of the factor structure of the DAQ have revealed that the factor structure is inconsistent across studies. For example, Kramer et al. (2010) reported three factors: strong desires/intentions to drink, negative reinforcement, positive reinforcement and the ability to control drinking, whereas Pasche et al. (2013) reported three different factors: desire to drink, ability to control drinking and positive and negative reinforcements. For a review of the issues in the measurement of craving, see Kavanagh et al. (2013). Given the inconsistency in the factor structure of the scale, we analysed the higher order factor (the mean scale score).

#### Subjective intoxication scales (Duka et al. 1998)

The Subjective intoxication scales (SIS) consisted of six 1–10 cm Likert scales (strongly disagree to strongly agree) which assessed subjective feelings of ‘lightheaded’, ‘irritable’, ‘stimulated’, ‘alert’, ‘relaxed’ and ‘contented’.

### Taste test

The taste test was based on that described previously (Field and Eastwood 2005). Participants were given 275 ml of Becks non-alcoholic beer and 275 ml of orange flavoured drink (a non-alcoholic beverage) served in glasses. Participants were not informed that the beer was non-alcoholic. We elected to use a non-alcoholic beer because previous studies from our laboratory have shown that participants do not detect that this brand of beer is non-alcoholic, and it has been used in previous taste test procedures which were sensitive to experimental manipulations of the motivation to drink (e.g. Christiansen et al. 2013; Fernie et al. 2012; Jones et al. 2011). Participants were asked to taste the two drinks and rate them on ten factors (e.g. How sweet is the drink?) using 10-point Likert scales. Participants were informed that they were allowed to drink as much of each drink as they wished in order to make ratings. At the end of the session, the volume of each drink consumed was recorded. We used the percentage of beer out of the total liquid consumed as the dependent variable (Christiansen et al. 2013). Informal debriefing indicated that none of the participants were aware that the beer was non-alcoholic.

## Procedure

Testing sessions took place between 12 p.m. and 6 p.m. in either a bar laboratory or a standard laboratory in the Department. The bar laboratory is a semi-naturalistic setting which includes all standard bar paraphernalia, including a well-stocked refrigerator, spirit optics, bar stools and seating arrangements similar to that found in a British pub. The standard laboratory consisted of a small testing room containing a desk with a computer and chairs for the participants and experimenter only. Participants were informed that the study was an investigation into the effects of alcohol on taste perception, and it was therefore important to abstain from drinking alcohol before each session and to avoid heavy drinking the night before. All participants provided a zero breath alcohol reading before each session (Lion Alcometer 500, Lion Laboratories, UK). Firstly, participants completed a battery of questionnaires (demographics, the TLFB and AUDIT). Participants then immediately completed the DAQ and SIS questionnaires. Dependent on condition, participants were then served either the placebo or control drink and were instructed to consume the drink within 10 minutes. To corroborate the cover story, participants were provided with magazines to read for 10 minutes and told that this period was necessary for absorption of their drink. After this ‘absorption’ period, participants completed a further DAQ and SIS questionnaire and provided a breath alcohol sample which the experimenter pretended to make a note of (all samples were .00 BAC). Participants then estimated how many standard UK units of vodka (25 ml) they believed were in the drink, before completing the taste test. Each testing session lasted approximately 40 min, and at the end of the second session, informal debriefing indicated that no participants had guessed the aims of the experiment.

## Statistical analysis

Before analysis, a square root transformation was conducted on all variables to ensure they met parametric assumptions; however, raw means are provided below for description. Data were analysed using mixed ANOVA. For the investigation of the effect of drink type and laboratory type on craving and light-headedness, these analyses had one between-subject factor (bar lab, standard lab) and within-subject factors of time (pre-drink, post-drink) and drink (placebo, control). For the analysis of beer consumed in the taste test, there was one between-subject factor (bar lab, standard lab) and a within factor of drink (placebo, control).

## Results

### Sample characteristics (see Table 1)

Participants were heavy drinkers consuming an average of 22.74 UK units per week with mean AUDIT scores of 12.78. Critically, participant characteristics were not different across the between-subject condition. It was notable that three participants believed that the placebo drink did not contain alcohol; removing these participants had no effect on the pattern of results reported in Table 1 (data available on request).

**Table 1:** Demographics and alcohol use indices for the whole sample and split by condition (values are mean ± SD)

	Sample (*n* = 64)	Bar lab (*n* = 32)	Standard lab (*n* = 32)	*t*(62)	*p*
Gender (F:M)	53:11	27:5	26:6	*χ* ^2^ = .11	.74
Age	19.54 (±3.50)	19.92 (±4.90)	19.20 (±1.37)	0.78	.44
Alcohol cons.	22.74 (±14.03)	24.09 (±14.79)	21.39 (±13.31)	0.77	.44
AUDIT	12.78 (±4.78)	12.53 (±4.97)	13.03 (±4.65)	−0.05	.62

Alcohol cons. = 7 day alcohol consumption in UK units, 1 unit = 8 g alcohol; AUDIT = Alcohol Use Disorders Identification Test, possible range of scores is from 0 (minimum) to 40 (maximum). Males and females did not significantly differ on age or any alcohol use measure, data available on request. Statistical significance refers to the lab comparison

### Subjective intoxication (Table 2)

There was a significant drink × time interaction on subjective feelings of light-headedness, reflective of a significant increase in lightheaded ratings from pre-drink to post-drink in the placebo condition (*p* < .001), but no significant change in the control condition (*p* > .1). There was no effect of lab type. Consistent with previous studies (Chutuape et al. 1994; Rose et al. 2014; Christiansen et al. 2016), other measures of intoxication were not affected by the placebo (data not reported but available on request). There was however a significant main effect of setting on *contented* with participants reporting feeling more contented on all assessments in the bar laboratory compared to the standard laboratory *F* (1, 61) = 17.22, *p* < .001, *η*_p_^2^ = .22.

**Table 2:** Descriptive statistics for craving, lightheaded ratings and beer consumed in the taste test (values are mean ± SD)

	Sample (*n* = 64)				Bar lab (*n* = 32)				Standard lab (*n* = 32)					
	Placebo		Control		Placebo		Control		Placebo		Control		F (1, 62), (*η*_p_^2^)	
	Pre-drink	Post-drink	Pre-drink	Post-drink	Pre-drink	Post-drink	Pre-drink	Post-drink	Pre-drink	Post-drink	Pre-drink	Post-drink	Time × drink	Time × drink × lab
DAQ	2.41 (0.95)	2.71 (1.02)**	2.52 (0.78)	2.48 (0.84)	2.31 (0.95)	2.72 (0.99)	2.41 (0.72)	2.36 (0.79)	2.50 (0.95)	2.70 (1.06)	2.64 (0.83)	2.61 (0.88)	18.65** (0.23)	2.33 (0.04)
Lightheaded	0.70 (1.27)	1.44 (1.64)*	0.98 (1.73)	0.97 (1.57)	0.50 (1.21)	1.58 (1.73)	0.81 (1.40)	0.88 (1.41)	0.91 (1.31)	1.29 (1.56)	1.15 (2.01)	1.06 (1.74)	13.98** (0.18)	2.30 (0.04)
													Drink	Drink vs. Lab
Taste Test	–	50.36 (20.52)	–	44.13 (22.83)*	–	51.57 (21.28)	–	42.13 (24.51)	–	49.14 (19.99)	–	46.14 (21.22)	4.47* (0.07)	1.23 (0.02)
Unit est	–	1.15 (0.52)	–	0.00 (0.00)**	–	1.29 (0.32)	–	0.00 (0.00)	–	1.01 (0.31)	–	0.00 (0.00)	326.78**(0.84)	4.64 (0.07)*

DAQ = mean scores on Desires for Alcohol Questionnaire, score range from 1(minimum) to 7(maximum). Subjective intoxication lightheaded scores, scores range from 0 (minimum) to 10 (maximum). Taste test = beer consumed as % of total liquid consumed. Unit est = number of 25 ml vodka measures (1 measure = 8 g of alcohol, one UK unit) participants believed to be in the priming drink. Statistical significance demoted for paired sample planned comparisons and F values

*p < .05; **p < .01

### Craving (Table 2)

#### DAQ

There was a significant drink by time interaction. Craving did not change after the control drink (*p* > .1), but it significantly increased following the placebo (*p* < .001); lab type did not affect these results.

### Taste test (Table 2)

There was a significant main effect of drink on percentage of beer consumed in the taste test, with a greater percentage of beer consumed in the placebo compared to the control condition (*p* = .039). There was no two-way interaction between drink and laboratory.

### Unit estimate (Table 2)

All except three participants reported having consumed alcohol in all of the placebo sessions (range from 12.5 to 75 ml), as opposed to none of the control sessions, resulting in a large main effect of drink type (*p* < .001). This effect was, however, moderated by context, with higher estimates in the bar lab compared to the standard lab (*p* = .035).

## Discussion

In the current study, we investigated the anticipated effects of alcohol on craving, subjective intoxication and voluntary beer consumption, and whether environmental context (standard vs. bar laboratory) and drinking status would moderate these effects. We hypothesised that the placebo would increase craving, beer consumption and subjective intoxication, with these effects being greater in the bar laboratory. The results partially supported our hypotheses. We found that craving, subjective intoxication and beer consumed, was increased by the placebo, but these effects were not dependent upon environmental context. Environmental context only significantly influenced perception of number of units consumed in the placebo drink with those in the bar lab believing that they had consumed more.

Importantly, we found that placebo-alcohol led to significant increases in alcohol craving, subjective ratings of light-headedness and beer consumed in a taste test. Previous studies have revealed that a placebo can increase craving and light-headedness (Christiansen et al. 2016; Christiansen et al. 2013; Rose et al. 2014) and voluntary beer consumption (Marlatt et al. 1973). Furthermore, results are also consistent with demonstrations that increased consumption of drinks believed to contain alcohol (irrespective of actual content) is not affected by environmental context (Wall et al. 2001). Taken together, these findings highlight the importance of anticipated effects of alcohol on the alcohol priming effect. Recent studies investigating the priming effect have demonstrated the pharmacological effects on the alcohol priming effect (e.g. Fernie et al. 2012; Weafer and Fillmore 2008), but due to the lack of an adequate control condition, they would have been unable to capture the anticipated effects of alcohol on this measure. This is an important limitation, as a real world priming effect is the combination of the anticipated and pharmacological effects of alcohol, so much previous research lack ecological validity. Moreover, there is evidence that the anticipated effects of alcohol can influence implicit (e.g. Christiansen et al. 2013) and executive (e.g. Christiansen et al. 2016) cognitive processes that are implicated in problematic alcohol consumption (e.g. Wiers et al. 2007) and the priming effect (Field et al. 2010). The lack of adequate control conditions in priming studies is therefore also limiting our understanding of the psychological mechanisms that underlie the priming effect.

Taken together, our findings suggest that experimental investigations of the alcohol priming effect should utilise a control condition and also consider the moderating influence of environmental context. Researchers should also be aware that the studies that omit the additional control beverage (to focus only on alcohol-placebo comparisons) will not fully capture how the alcohol priming effect is likely to operate in naturalistic settings.

It is notable that the environmental context seems to have little effect apart from the finding that estimations of the amount of alcohol in the placebo drink were increased by naturalistic settings. Previous studies have found that (in the absence of an alcohol or placebo prime) alcohol-related contexts such as bars can increase ad-lib consumption (Lau-Barraco and Dunn 2009; Moss et al. 2015, although see also Fromme and Dunn 1992). One tentative explanation for this is that the effect of placebo-alcohol is comparatively large, and the more subtle effects of environmental cues do not have an additive influence on this placebo effect. Despite this, when asked to estimate how many units of alcohol were consumed in the placebo condition, participants made greater estimations in the bar lab suggesting some contextual influence is apparent following a placebo. This may be the result of participant making estimates of units consumed informed by long term memories of past behaviours (“when in a bar I usually drink doubles”), rather than merely reporting a current subjective experience as is the case for intoxication and craving.

Our findings are important because some theoretical models suggest that increased ad-lib alcohol consumption following an alcohol prime is due to alcohol-induced impairments in executive cognitive functioning, increases in the incentive motivational properties of alcohol, or a combination of the two (Field et al. 2010; Koob and Le Moal 2001; Wise and Bozarth 1987). However, Moss and Albery’s (2009) dual process model integrating alcohol myopia and expectancy theory can account these findings. Within the context of this theory, it could be argued that the consumption of placebo-alcohol functions as a compound conditioned stimulus activating alcohol-related expectancies and subsequent behaviours thereby driving alcohol consumption. This emphasises the importance of the pre-consumption phase of drinking that is ignored by many other models of alcohol consumption.

The current study has several limitations. Most importantly, our sample was predominantly female, and, for practical reasons, participant allocation to the standard or the bar lab in counterbalanced blocks rather than random (although groups were balanced on critical characteristics). The gender imbalance makes it difficult to extrapolate findings to alcohol-dependent populations, who are predominantly male. Running lab conditions sequentially is not ideal, but identical recruitment techniques were used; lab testing took place at exactly the same time of year, and participants were matched on demographic variables and alcohol use. We encourage replications of our findings in more diverse populations with larger samples.

In conclusion, the current study demonstrated the importance of the anticipated effects of alcohol and alcohol-related contexts on craving and ad-lib drinking. This has important implications, because it suggests that studies that attempt to characterise the alcohol priming effect may be missing out on important aspects of the process and limiting their validity as a consequence.

## Electronic supplementary material

ESM1 (DOCX 11 kb)
