# Supplementary material for: Bibi ergo sum: the effects of a placebo and contextual alcohol cues on motivation to drink alcohol
Source: Psychopharmacology (Berl). 2017 Jan 7;234(5):827–35. doi: 10.1007/s00213-016-4518-0 (PMC5306434; doi:10.1007/s00213-016-4518-0)
Supplement: Supplementary file 1 — (DOCX 11 kb) [file 213_2016_4518_MOESM1_ESM.docx]

Supplementary analysis: Female only sample

Due to the limited number of males we reanalysed the data with female participants only (N=53). Results for craving and light-headed are consistent with that of the analysis conducted in the full sample, although results for the effect on ad lib consumption are different, with the effect of drink being no longer significant (although it was in males only).

With regards to craving there was a significant time by drink interaction F(1, 51)=19.99, p<.001, η_p_^2^=.25, (caused by significant increases in craving following the placebo drink only), but no drink by time by setting interaction F(1, 51)=1.82, p=.18, η_p_^2^=.03. Similarly, for light-headedness there was a significant time by drink interaction F(1, 51)=6.02, p=.003, η_p_^2^=.17, (caused by significant increases in light-headedness following the placebo drink only), but no drink by time by setting interaction, F(1, 51)=3.53, p=.055, η_p_^2^=.07. Finally, the significant main effect of drink found for in taste that was is not apparent in the female only sample F(1, 51)=1.50, p=.22, η_p_^2^=.03.
